# Ageing and Caloric Restriction in a Marine Planktonic Copepod

**DOI:** 10.1038/srep14962

**Published:** 2015-10-12

**Authors:** Enric Saiz, Albert Calbet, Kaiene Griffell, José Guilherme F. Bersano, Stamatina Isari, Montserrat Solé, Janna Peters, Miquel Alcaraz

**Affiliations:** 1 Institut de Ciències del Mar – CSIC, Pg. Marítim de la Barceloneta 37-49, 08003 Barcelona, Catalonia, Spain; 2 Centro de Estudos do Mar, Universidade Federal do Paraná, Pontal do Paraná, PR, Brasil; 3 Institute of Oceanography, Hellenic Centre for Marine Research, P.O. Box 712, 19013 Anavyssos, Athens, Greece; 4 Institute for Hydrobiology and Fisheries Science, Hamburg University, Grosse Elbstrasse 133, 22767 Hamburg, Germany

## Abstract

Planktonic copepods are a key group in the marine pelagic ecosystem, linking primary production with upper trophic levels. Their abundance and population dynamics are constrained by the life history tradeoffs associated with resource availability, reproduction and predation pressure. The tradeoffs associated with the ageing process and its underlying biological mechanisms are, however, poorly known. Our study shows that ageing in copepods involves a deterioration of their vital rates and a rise in mortality associated with an increase in oxidative damage (lipid peroxidation); the activity of the cell-repair enzymatic machinery also increases with age. This increase in oxidative damage is associated with an increase in the relative content of the fatty acid 22:6(n-3), an essential component of cell membranes that increases their susceptibility to peroxidation. Moreover, we show that caloric (food) restriction in marine copepods reduces their age-specific mortality rates, and extends the lifespan of females and their reproductive period. Given the overall low production of the oceans, this can be a strategy, at least in certain copepod species, to enhance their chances to reproduce in a nutritionally dilute, temporally and spatially patchy environment.

Copepods are small crustaceans that comprise the major part of zooplankton biomass in the oceans, and they probably constitute the most abundant metazoan fauna on the planet^1^. Particular morphological, physiological and behavioural traits have made them a very successful group in the pelagic realm^2^, with a key role in the transfer of primary production to upper trophic levels and thus in the general functioning of the marine ecosystem^3^,^4^. Traditionally, predation has been considered a major source of mortality in marine copepods^5^,^6^. Recent studies, however, suggest that non-predatory mortality might be much more important than previously thought, accounting for 25–33% of total mortality of copepods at a global scale^7^. That agrees with the high abundance of copepod carcasses often found in the oceans^8^,^9^,^10^. Parasites, together with natural intrinsic mortality (i.e. age-related), are considered relevant although poorly quantified components of this non-predatory mortality^11^,^12^,^13^.

The ageing of individuals, defined as the progressive deterioration of physiological functions and the associated increase in age-specific mortality, can certainly influence not only individual performance, but also the population dynamics and evolution of life history traits in marine copepods^14^,^15^,^16^. In contrast to other animal groups, like rotifers, cladocerans, nematodes, flies, birds and mammals^17^,^18^,^19^,^20^,^21^, the biology of ageing, its underlying mechanisms and the implications for life history theory have seldom been studied in copepods^22^,^23^. For this reason, the ageing of copepods has rarely been taken into consideration in individual-based, population or biogeochemical-flux modelling^24^,^25^.

In copepods, the cost of mating has been associated with a reduction in the lifespan of females and males^15^,^26^,^27^,^28^; both female fecundity and male mating capacity also decrease with age^15^,^27^,^29^,^30^. Presumably, those observations can be included within the framework of the general theory of ageing, and the shorter life expectancy could be interpreted as the result of an increase in oxidative stress^31^,^32^, as in other animals. However, this issue has never been addressed in copepods. The longevity and ageing patterns of several species of marine copepods in the laboratory, in the absence of extrinsic mortality, have been linked recently to tradeoffs associated with species-specific feeding and mate-finding behaviours, as well as to spawning behaviours^14^. The trade-offs between reproductive effort and survival, however, can be complex, and often the proximate causes of reproductive costs are not well known^14^,^33^. Information on other aspects of the ageing process in copepods (e.g., changes in feeding, routine metabolism, oxidative damage, antioxidant and repair capacities) is scarce or even nonexistent^34^,^35^. Only a study by Rodríguez-Graña *et al*.^35^ has attempted to link oxidative damage and ageing in copepods. They found an increase in the levels of carbonylated proteins (a biomarker of oxidative damage to proteins) with age in males of the copepod *Acartia tonsa*, but they found no such variation in females^35^. Despite the latter result, current knowledge of the biology of ageing supports the view that a certain level of reactive oxygen species (ROS) is always present and is essential to maintaining homeostasis in animals. It appears that shifts in the delicate balance among ROS generation, antioxidant defences and cell-repair systems might be the links among oxidative damage, ageing and lifespan^18^,^36^,^37^.

Here we report results from a thorough study of some of the processes associated with ageing in the marine calanoid copepod *Paracartia grani*, which belongs to one of the most frequent copepod families found in estuaries and coastal waters around the world. We have assessed changes in somatic elemental/biochemical composition and in vital rates of females throughout their adult lifespan. The senescence patterns observed were correlated with an increase in the amount of oxidative damage, along with increased activity of oxidative-stress repair mechanisms. Moreover, we provide empirical evidence that caloric restriction, defined as a reduction in food consumption in comparison to satiation, and therefore encompassing not only a reduction in the intake of calories but also in all macro and micronutrients^38^, extends the lifespan in copepods as occurs in many other animals^38^. This likely is a life-history strategy to ensure reproductive investment in the ocean, a nutritionally dilute, temporally and spatially patchy environment^39^,^40^.

## Results

### Changes in chemical composition and vital rates with age

**Figure 1:**
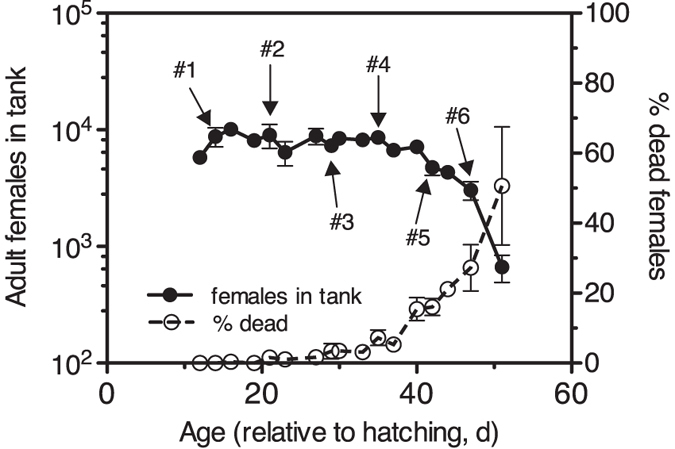
Tank experiments. Time series of the abundance and mortality of females. The abundance has been corrected for the extraction of individuals due to sampling and experiments. Numbers indicate the dates of the vital rate incubations. Mean ± s.e.m. (n = 3).

Even-aged cohorts of female *P. grani* reared in tanks and fed high concentrations of the alga *Rhodomonas salina* remained stable in numbers up to day 40 of their life (Fig. 1). Abundance decreased progressively after then. This decline was parallel to an increase in the presence of recently deceased females in the tanks (up to 50% by the end of the experiment; Fig. 1).

**Figure 2:**
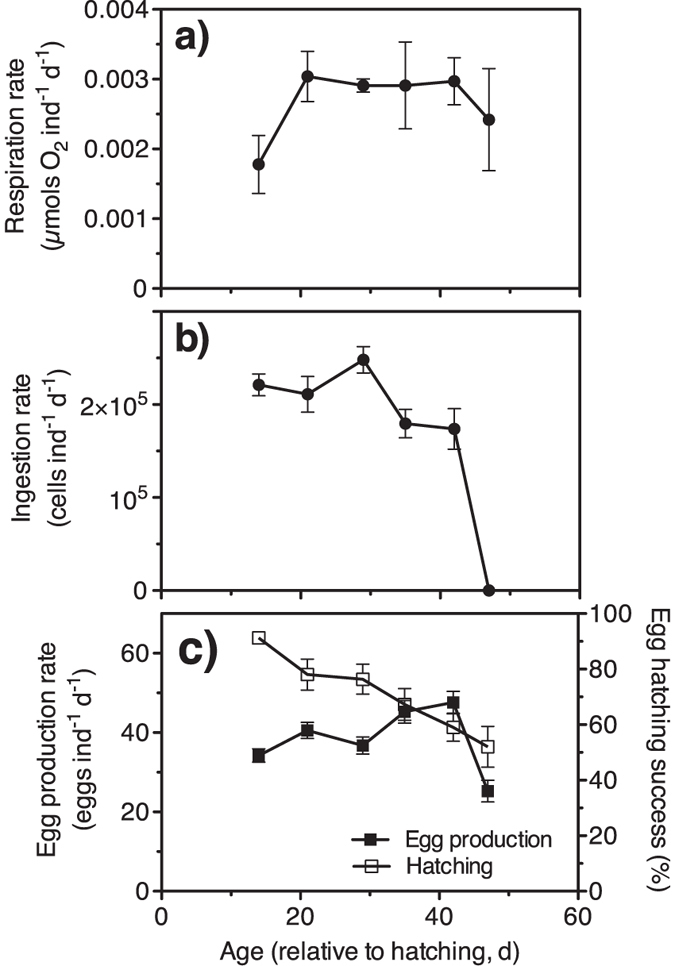
Tank experiments. Changes in the vital rates (mean ± s.e.m.) of females with age. (**a**) Respiration rate (n = 3–6 chambers); (**b**) Ingestion rate (n = 6 bottles); (**c**) Egg production rate (n = 30–36 females held singly) and hatching success (n = 22–35 egg batches).

We assessed the variation in the vital rates (respiration, feeding, egg production, and egg hatching success) of the females in the tanks on six occasions across the adult lifespan (Fig. 1). Respiration rates of adult females increased ca. 50% after the first week and then remained relatively constant until the end of the tank experiments (Fig. 2a), when the death of the population was nearly complete. Feeding rates of *P. grani* females were quite stable for the first weeks of adulthood, started to decline on day 30 and then dropped suddenly before the monitoring ended (Fig. 2b). The last assessment detected no significant ingestion of cells, despite the experimental copepods still being alive at the end of the incubation. Additional feeding incubations with fresh, old females conducted a day later further confirmed this lack of feeding.

Egg production rates of *P. grani* increased with age for the first weeks of adulthood, reaching ca. 50 eggs ind^**−**1^ d^**−**1^; then egg production rates during the last week decreased to ca. 25 eggs ind^**−**1^ d^**−**1^ (Fig. 2c). Egg hatching success declined steadily with age from ca. 90% in freshly moulted females to ca. 50% in the oldest females (Fig. 2c).

**Figure 3:**
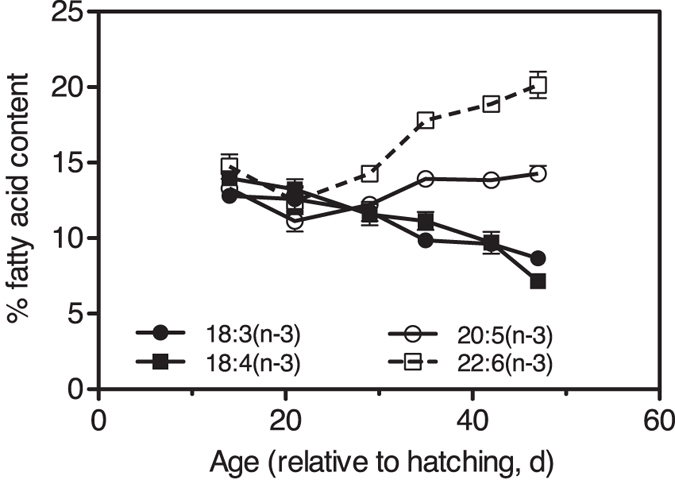
Tank experiments. Changes in the relative contents of the main polyunsaturated FAs in females with age. Mean ± s.e.m. (n = 3).

Elemental composition of *P. grani* females was quite stable throughout the experiment, although the carbon and phosphorus contents decreased with age by 21% and 10%, respectively (linear regression analysis: C: r^2^ = 0.53 and p < 0.008, P: r^2^ = 0.39 and p < 0.032, n = 12; Table 1). Stoichiometry (molar ratios) was also rather stable through the female lifespan, except for C/N, which decreased with age by 11% on average (C/N: r^2^ = 0.58 and p < 0.004, n = 12; Table 1). Regarding fatty acid (FA) composition, we observed significant decreases in the contributions of 18:3(n-3) and 18:4(n-3) FAs with age (linear regression analysis: respectively r^2^ = 0.79 and r^2^ = 0.83, in both cases p < 0.001 and n = 18; Fig. 3), and significant increases with age in the contributions of the FAs 20:5(n-3) and particularly 22:6(n-3) (linear regression analysis: respectively r^2^ = 0.32 and p < 0.016, and r^2^ = 0.69 and p < 0.001, n = 18; Fig. 3).

We determined the activity of enzymes related to oxidative-damage repair, and we quantified the amount of oxidative stress damage as the levels of lipid peroxidation (LPO) through the progression of the adult female lifespan. The activities of glutathione S-transferase (GST) and catalase (CAT) increased with age (linear regression analysis: GST: r^2^ = 0.31 and p < 0.016, CAT: r^2^ = 0.54 and p < 0.001, n = 18; Table 2). The LPO level also varied, although the positive linear relationship was not statistically significant (r^2^ = 0.19 and p = 0.070, n = 18; Table 2). Nevertheless, if we compare the first and the last 3-week periods of adult longevity, LPO levels were 69% higher shortly before death (one-tailed t-test, p < 0.018). The GST and CAT activities and LPO levels were all positively correlated with the mortality rate (% dead females, after arcsine transformation) in the tanks (GST: r = 0.483, p < 0.043; CAT: r = 0.579, p < 0.012; LPO: 0.553, p < 0.018; n = 18).

### Effects of caloric restriction on *P. grani* females

**Figure 4:**
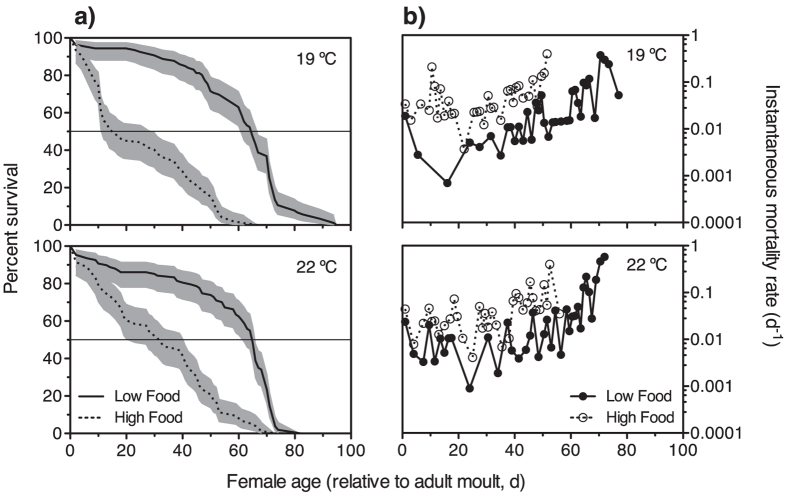
Caloric restriction experiments. Kaplan-Meier survival curves (**a**) and temporal variation in age-specific instantaneous mortality rate (**b**) for females under high and low food supplies at 19° and 22 °C. 95% confidence intervals are shown for the survival curves.

**Figure 5:**
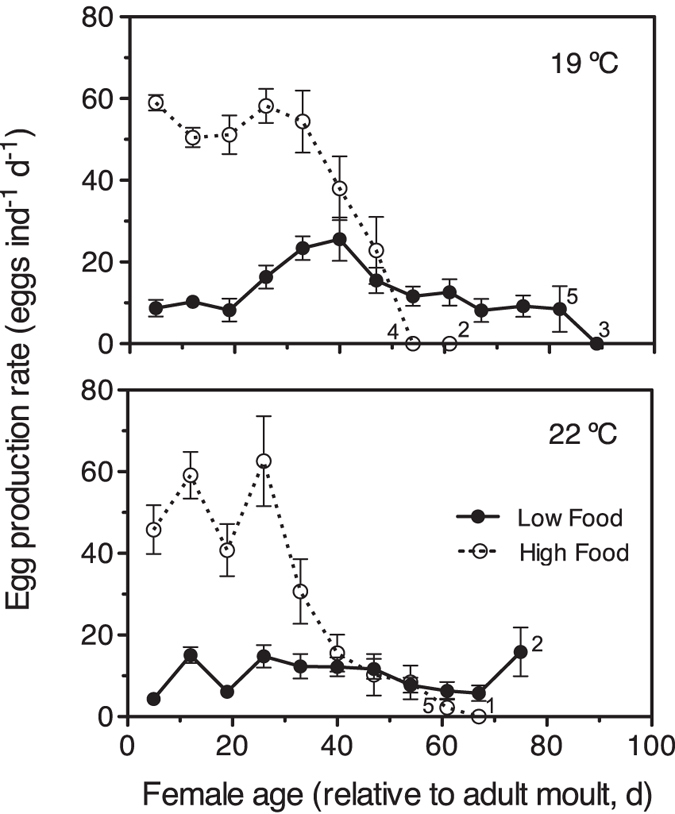
Caloric restriction experiments. Variation in egg production rates with age under high and low food supplies at 19° and 22 °C. Mean ± s.e.m. Sample size was 10–12 females held singly, except for the determinations at the very end of the experiment (see numbers in graph).

We assessed the survival of females exposed to high and low food rations by monitoring individual females from the time they entered the adult stage until death. At 19 °C and 22 °C, caloric restriction (food-limiting conditions) significantly extended the survival of females (Fig. 4a; Log-rank and Gehan-Breslow-Wilcoxon tests, p < 0.001 and p < 0.001, respectively). Median survival times at 19 °C were 65 (95% CI: 61–67) and 16 (95% CI: 12–30) days at low and high food concentrations, respectively; the ratio between median survival times was 4.063 (95% CI of ratio: 3.303 to 4.822). At 22 °C the median survival times at low and high food concentrations were, respectively, 65 (95% CI: 62–66) and 33 (95% CI: 23–41) days; the ratio between median survival times was 1.970 (95% CI of ratio: 1.208 to 2.732). Life expectancies at low and high food concentrations, respectively, were 58.7 and 24.4 days at 19 °C, and 54.8 and 31.0 days at 22 °C. The age-specific instantaneous mortality rate increased with age and was lower overall under low food concentrations (Mann-Whitney tests, p < 0.006 and p < 0.013 for the 19 °C and 22 °C treatments, respectively; Fig. 4b). Weekly determinations of egg production rates (Fig. 5) showed that adult females under limiting food conditions were able to spawn almost to the ends of their lives (week 12^th^ after adulthood), although at much lower rates. In the 19 °C treatment, the egg production rates (mean ± s.e.m., only observations with >10 females) under satiating and food-restricted conditions were, respectively, 48 ± 4.9 (n = 7) and 14 ± 1.8 (n = 11) eggs ind^**−**1^ d^**−**1^; differences between paired data were statistically significant (paired t-test, two-tailed p < 0.003, n = 7; Fig. 5). At 22 °C, the egg production rates under satiating and food-restricted conditions were, respectively, 34 ± 7.6 (n = 8) and 10 ± 1.3 (n = 10) eggs ind^**−**1^ d^**−**1^; differences between paired data were statistically significant (paired t-test, two-tailed p < 0.015, n = 8; Fig. 5). Throughout their lifespans the accumulated egg production totals were 1,015 and 2,462 eggs per female at 19 °C, and 684 and 1,959 eggs at 22 °C per female in the low and high food treatments, respectively.

When planning the caloric restriction experiments at two temperatures we anticipated that effects at the highest temperature might be stronger, in particular regarding the median survival times. However, in the low-food treatment the ratio between the median survival times at 19 °C and 22 °C was 1.00 (95% CI of ratio: 0.238 to 1.762); in the high-food treatment the ratio between the median survival times at 19 °C and 22 °C was lower, 0.485, but still not significantly different from 1 (95% CI of ratio: −0.275 to 1.245). The lack of substantial significant differences between temperatures in the median survival times at a given food concentration most likely lies in the fact that the degrees of food satiation and food restriction were not comparable, since at the higher temperature metabolic requirements would have been greater, and the same food concentration could not provide equally for metabolic demands.

## Discussion

To our knowledge, this study produced the most comprehensive data set regarding the changes in physiological rates associated with ageing in marine copepods. In general, our results agree with previous observations of age-related declines on copepod fecundity^30^,^34^,^35^ and feeding rates^34^,^35^. The observed pattern of decline in egg hatching success in our experiments, however, cannot be fully attributed to a decline in female fertility^29^,^30^, as the females were mixed with males while in the tanks and conditioning bottles, and changes in male mating capacity and fertility likely occur with age^15^,^27^,^30^. Along with the patterns of change in routine metabolism (respiration) and somatic elemental composition associated with age, *P. grani* females were largely able to maintain homeostasis and to sustain rather high feeding rates and reproductive activity until nearly the end of their lifespan.

Changes in the enzymatic complex involved in oxidative damage repair began early in the adult lifespan, and both CAT and GST increased with age. CAT is a key antioxidant enzyme, eliminating peroxides in the cell. GST is a detoxifying enzyme that catalyses the conjugation of glutathione that can then directly react with ROS or act as a co-substrate of glutathione peroxidases, another key group of antioxidant enzymes^18^,^37^. The production of ROS can adversely affect important biological molecules^17^. Among those, the oxidative damage of lipids (LPO) has serious consequences for membrane integrity and function^36^. Accumulating LPO is associated with the ageing of cells, and not only damages membrane lipids, but produces highly reactive molecules that can affect mitochondrial DNA and proteins^18^. In our study, the levels of LPO of *P. grani* adult females increased with age, particularly during the second half of their adulthood. During this period, mortality rates of copepods in the tank increased rapidly, coinciding with a decline in ingestion rates but not in fecundity. We also observed changes in the FA composition of females toward the end of their life, particularly an increase of the FA 22:6(n-3), a major component of cell membranes. We think that the increase in oxidative damage (in LPO levels) near the end of life in *P. grani* females is likely associated with this high relative content of FA 22:6(n-3), as reported for other animals^41^. The high degree of polyunsaturation of the cell membrane FA 22:6(n-3) makes it much more susceptible to free radical attack^18^. Thus, ageing cell membranes become particularly sensitive to attack by ROS and susceptible to progressive lipid peroxidation and oxidative damage^18^,^41^.

In our experiments, the antioxidant defence systems of *P. grani* females also increased with age, likely counteracting the oxidative damage until physiological functions were compromised leading to death. These observations could be interpreted within the framework of the “traditional” free radical or oxidative stress theory of ageing, the notion that oxidative stress increases progressively with age mainly due to the generation of ROS and, to a lesser extent, to a decline in antioxidant defences. The eventual result is loss of homeostasis^18^,^31^,^42^. Although the universality of this theory has been questioned^31^,^38^,^43^, it remains robust. It is being reformulated to accommodate more contemporary understanding, including the key physiological regulatory and signalling function that certain ROS may have at relatively low concentrations and the relationship between elevated oxidative stress and age-related disease^42^,^44^.

In both our tank experiments and our caloric restriction experiments *P. grani* females were spawning until close to the time of death. Similarly long reproductive periods have been also found for the copepods *A. tonsa*^29^,^35^, *Centropages typicus*^13^,^34^ and *Eurytemora affinis*^28^,^45^. On the contrary, short reproductive periods of mated females relative to their lifespans have been reported for *Oithona davisae* (12–16 days in a 40–70 day longevity)^15^ and *Temora longicornis* (14 days in a 30 day longevity)^15^,^30^. In the absence of parental care or social defence benefits, the reasons that some groups of organisms exhibit prolonged post-reproductive lifespans (PRLS) are, from an evolutionary point of view, a challenge^46^,^47^. Life history theory does not predict the existence of PRLS, and it is rather likely that the long PRLS observed in certain copepod species does not really appear in nature, as females might not live so long under high predation^23^. Further understanding of the mechanisms and tradeoffs regulating the duration of the fertile period and PRLS in pelagic copepods is required, as well as application of biochemical or molecular techniques allowing the assessment of age-structure in wild copepod populations.

It has long been recognized that restricting food intake in the laboratory can extend lifespan in a wide range of animals^48^,^49^. Our study provides the first evidence that caloric (food) restriction may cause a change in copepod survival curves by lowering age-specific mortality rates and increasing life expectancy. Though there are exceptions and criticisms, caloric restriction is accepted as a nearly universal paradigm, evolutionarily preserved to modulate the intrinsic rate of ageing among animals^38^,^42^. The relationship between caloric restriction and longevity may not necessarily be linked to metabolism as formerly thought; specific underlying mechanisms, similar to those in the ageing process but yet not well understood, could attenuate the overall effects of oxidative stress^38^,^42^. A broader framework including other factors, such as hormetic effects, have been proposed to explain the enhanced defence mechanisms and increased longevity under caloric restriction^32^,^48^,^49^.

One of the mechanisms by which caloric restriction could affect lifespan is restraint on reproductive effort. Egg production is energetically demanding and, according to the life history theory, high investment in reproduction reduces the chances of survival in many animals^33^, including copepods^15^,^26^,^27^,^28^. In our caloric restriction study, the life long, cumulative egg production at low food concentration was much lower, despite the extended lifespan, than at high food concentration. At 19 °C the accumulated reproductive effort until the end of life at the restricted food ration was 2.4-fold less than at high food ration, whereas life expectancy was increased by the same factor, suggesting a coupling between survival and reproductive costs. The additional reproduction during the extended lifespan under food-restricted conditions, however, was not able to reach even close to the total egg production realized under the shorter-lifespan, food-satiated conditions, likely due to the soma maintenance costs at low food availability. The disposable soma theory postulates tradeoffs between somatic maintenance costs and reproductive costs; hence, an individual with greater food resources does not necessarily invest the energy gain to live longer, but may obtain a higher reproductive output within a shorter time^50^. At 22 °C, the coupling between survival and the cost of reproduction was less tight, since the corresponding factors in cumulative egg production and life expectancy were 2.9-fold and 1.8-fold, respectively. The amount of food offered to copepods on low rations was likely insufficient to cope with the faster metabolism at higher temperature.

The response to caloric restriction can be interpreted as an evolutionary strategy, one ensuring survival through periods of food shortage to give at least some offspring a chance for recruitment^50^. Optimal allocation of resources, however, may change with age as a result of life history tradeoffs associated with changes in environmental conditions (i.e. food, predation risk) and reproductive, foraging and survival costs^51^. Our results suggest that the patterns of senescence and mortality in copepod populations may be influenced by resource availability during their life cycle^51^.

## Materials and Methods

### Experimental organism

Copepods used in the study came from a culture of *P. grani* kept in our laboratory at 19 °C and fed the alga *Rhodomonas salina*. This copepod culture originated from wild specimens collected in coastal waters near Barcelona (NW Mediterranean) more than 7 years ago. The size (cephalothorax length) of adult female *P. grani* is ca. 1000 μm. The cryptophyte *Rhodomonas salina* is widely used in copepod culturing, is generally considered high quality food for copepods^52^ and is also a valuable source of antioxidants^53^.

### Development of copepod cohorts to assess ageing effects on adult females

Batches of 40,000 fresh eggs of *P. grani* were collected and seeded into each of three replicate 30-L tanks, filled with 0.1-μm filtered seawater (FSW). Each tank was submerged in a water bath adjusted at 20.5–20.7 °C by a TECO TC-20 water conditioner, in a cold room with 18:6 h light:dark cycle. The day after seeding we siphoned out unhatched eggs, and after mixing we withdrew a 100-mL sample to assess the initial abundance of nauplii in each tank (28,000–30,000 per tank). From then on, each tank was sampled 2–3 times a week (up to 1,000 mL by the end of the monitoring, when copepods were less abundant) to assess the abundance and life-stage composition of the cohorts.

Initially, food concentration in the tanks was set daily to 1,400 μg C L^**−**1^ of *R. salina* (1,400 μg C L^**−**1^ *R. salina* amounts ca. 27,000 cells mL^**−**1^). Every weekday, food availability was monitored with a Coulter Multisizer III particle counter fitted with a 100-μm tube, and fresh *R. salina* added up to desired food levels. As copepods grew, daily food levels were raised to ca. 3,300–3,600 μg C L^**−**1^. Ultimately, as the copepod populations declined, food supply was reduced to keep a quota of 6 μg C per adult through the experiment. On average, daily food stocks amounted ca. 2,400 μg C L^**−**1^ (range: 1,100–3,700). On day 12 of the cohort development, adult males, adult females and C5 stages occurred in similar proportions (ca. 1/3 each). To avoid overlapping with new generations of copepods we transferred only adults (using a submerged 300-μm sieve) once a week to new tanks with *R. salina* suspensions.

### Mortality estimates

Adult mortality in the tanks was assessed by a modification of the neutral red method^54^. Dead (unstained) and live (stained) copepods from the tank were counted in a concentrated sample (ca. 20 mL) after 2 min staining period in neutral red solution (25 μg mL^**−**1^). Carcasses and decomposed specimens were not considered.

### General procedure for vital rate incubations

Every week we conducted incubations with females from the tanks to assess the changes in their respiration, feeding, egg production rates and egg-hatching success through their adult lifespans. Prior to that, in order to reduce potential bias due to variations in food availability among tanks, we conditioned 1,000 adult copepods from each tank for 48 h in 9-L Nalgene bottles (500 copepods bottle^**−**1^) filled with 1,100 μg C L^**−**1^ *R. salina* suspensions adjusted daily; this concentration is close to satiation values for this species (see supplementary information Figure S1 online). We also determined the individual C, N and P contents, FA composition, the activity of antioxidant enzymes, and LPO levels of females.

### Feeding incubations

Feeding rates of *P. grani* on the alga *R. salina* were obtained from cell-removal rates in bottle incubations assuming exponential algal growth and grazing. Conditioned *P. grani* females were picked out and incubated in 620 mL Pyrex bottles (8 copepods bottle^**−**1^) filled with fresh 1,100 μg C L^**−**1^ *R. salina* suspensions (2 bottles per replicate tank). Seven additional replicate bottles with only food suspension were prepared, three serving as initials and the others as control bottles. The bottles were left standing submerged inside the water baths where the tanks were located. Occasionally, the bottles were rotated end-over-end to homogenize their contents. After 24 h, their contents were filtered through a 200-μm sieve, females were checked and counted and food concentration was determined with the particle counter. Further details of the procedure can be found in Isari and Saiz^55^.

### Egg production

Conditioned females were transferred to 6-well plates, placing one single female in each well, previously filled with 9-mL of 1,100 μg C L^**−**1^ *R. salina* suspension. Two plates were set per replicate tank. After 24 h, the females were checked and removed, and the numbers of intact eggs and egg-shells (due to cannibalism) were counted. The plates were then left for an additional 48 h, and the numbers of unhatched eggs and nauplii were recorded. Egg production rates were based on the total number of eggs found at the end of the 24-h incubation. Hatching success was computed as the number of nauplii found after the hatching period and expressed as percentage of the intact eggs found after the 24-h incubation.

### Oxygen consumption

Groups of 20 conditioned females were incubated in respiration chambers (30-mL beakers) filled with FSW. Two replicate chambers with copepods were set-up per tank; four additional ones without copepods acted as controls. Each chamber was sealed by a silicone stopper with an inserted oxygen probe (PreSens). The chambers were then wrapped in aluminium foil and placed in the baths containing the copepod tanks. During the incubation (ca. 20 h), oxygen concentration measurements were taken every minute. The respiration rate was calculated as the difference in slope between the control and the experimental oxygen time series. To reduce the possible effects of starvation, respiration rates were computed based on the data for the first 8 hours (after discarding an initial 1-h stabilization period).

### CNP content and FA composition

Groups of either 15 (C and N, and P) or 30 (FA) live females from the conditioning bottles were transferred, after narcotizing with MS-222, onto pre-combusted 25-mm Whatman GF/C filters (450 °C, 6 h). Filters were dried for 24 h at 60 °C and kept in a desiccator until C and N analysis with a CHNS analyser. Filters for P content and for FA composition were frozen at −80 °C immediately after filtration. P content was analysed as orthophosphate after acid persulfate digestion. For the FA analysis, lipid extraction and conversion of acyl groups into FA methyl ester derivatives were conducted after Peters *et al*.^56^.

### Enzyme activity and LPO levels

Similarly to the procedure mentioned above, we transferred groups of 200 live females from the conditioning bottles onto small, 200-μm mesh filters and, after freezing them in cryovials submerged in liquid nitrogen, samples were stored at −80 °C until analysis. The methodology used to determine the GST and CAT activities, LPO levels, and total protein content is described in detail in Solé *et al*.^57^, and used an extraction procedure adapted from Yebra *et al*.^58^. The filters containing the copepods were sonicated in 500 μL of Tris buffer (20 mM at pH 7.8), the homogenate centrifuged at 10,000 g for 30 min at 4 °C, and the supernatant (S10) used for biochemical analysis. The total amount of protein in the homogenates was 2–3 mg mL^**−**1^. All assays were carried out in triplicate at 25 °C in 96-well plates using a Tecan Infinite M200 spectrofluorometer microplate reader. All activities were referred to total protein content.

### Caloric restriction experiments

We pre-sorted C5 females from a copepod cohort grown at 22 °C, and kept them in 2-L Pyrex bottles filled with a 2,200 μg C L^**−**1^ suspension of *R. salina*. After 1–2 days, four batches of ca. 108 newly moulted adult females were individually placed in 6-well plates (18 plates per batch); each female was provided 9 mL of either 800 or 2,700 μg C L^**−**1^ of *R. salina* and incubated at either 19 or 22 °C. From then on the survival and fecundity rates of females were followed until population death. We inspected female survival typically 5 times per week, and replaced 8 mL of fresh food suspension 3 times per week (Monday, Wednesday, and Friday); on Fridays, given the extra day over the weekend, the food supply was increased to 1,100 and 3,000 μg C L^**−**1^, respectively. To assess the variation of egg production with age, on Wednesdays we transferred 12 random females from each treatment to new plates with the respective food suspensions, and let them incubate for ca. 24 h; after that period, the females were returned to their original plates, and the eggs counted. The accumulated egg production throughout the lifespan was computed from the weekly average estimates, integrated up to the age at which survival rate of the population was 10% (to avoid highly variable numbers due to very few individuals at the tail of the survival curve). From previous data (see supplementary information Figure S1 online) and given that food was replenished every two-three days, we expected the experienced food rations at the low and high food treatments would be respectively rather limiting (but not starving) and satiated or close to satiation. The data on egg production from the caloric restriction experiments (see Results) confirmed this assumption when compared with the egg production functional response for this species^59^ (see supplementary information Figure S1 online).

Kaplan-Meier survival curves and statistical tests (Log-rank and Gehan-Breslow-Wilcoxon tests) were calculated with Prism 5.0f. The age-specific instantaneous mortality rates were estimated by the Cutler-Ederer hazard rate^19^. Life expectancy of adult females, defined as the average number of days to live after the onset of adulthood, was calculated as the area under the survival curve.

## Additional Information

**How to cite this article**: Saiz, E. *et al*. Ageing and caloric restriction in a marine planktonic copepod. *Sci. Rep*. **5**, 14962; doi: 10.1038/srep14962 (2015).

## Supplementary Material

Supplementary Information

## Figures and Tables

**Table 1:** Carbon, nitrogen and phosphorous contents, and molar ratios of *P. grani* females along the lifespan.

Day	μg C fem^−1^	μg N fem^−1^	μg P fem^−1^	C/N	N/P	C/P
14	7.4 ± 0.25	1.7 ± 0.06	0.23 ± 0.020	5.2 ± 0.02	16 ± 0.8	82 ± 4.3
21	7.7 ± 0.35	1.7 ± 0.09	0.22 ± 0.002	5.2 ± 0.03	17 ± 0.7	90 ± 3.1
29	7.0 ± 0.45	1.6 ± 0.11	0.22 ± 0.002	5.2 ± 0.02	16 ± 1.2	81 ± 6.0
35	6.6 ± 0.04	1.7 ± 0.05	0.20 ± 0.024	4.4 ± 0.09	19 ± 2.8	86 ± 10.7
42	5.6 ± 0.00	1.4 ± 0.04	0.20 ± 0.001	4.5 ± 0.13	16 ± 0.5	71 ± 0.2
47	6.3 ± 0.85	1.6 ± 0.17	0.20 ± 0.005	4.7 ± 0.11	17 ± 2.3	81 ± 12.7

Time is relative to the hatching of the cohort. Mean ± s.e.m. (n = 2).

**Table 2:** Activity of the enzymes glutathione S-transferase (GST; nmol min^ −1^ mg prot^ −1^) and catalase (CAT; μmol min^ −1^ mg prot^ −1^), and levels of LPO (equi MDA mg prot^ −1^) as a function of age in adult female *P. grani*.

Day	GST	CAT	LPO
14	2,505 ± 167	7.8 ± 0.69	0.50 ± 0.039
21	3,359 ± 318	9.9 ± 1.05	0.49 ± 0.249
29	2,718 ± 69	9.8 ± 0.33	0.42 ± 0.113
35	3,265 ± 289	11.8 ± 1.35	0.80 ± 0.179
42	3,622 ± 477	12.5 ± 1.34	0.64 ± 0.078
47	3,767 ± 462	12.9 ± 1.30	0.94 ± 0.312

Time is relative to the hatching of the cohort. Mean ± s.e.m. (n = 3).
