# Supplementary material for: Ageing and Caloric Restriction in a Marine Planktonic Copepod
Source: Sci Rep. 2015 Oct 12;5:14962. doi: 10.1038/srep14962 (PMC4601087; doi:10.1038/srep14962)
Supplement: Supplementary Information [file srep14962-s1.pdf]

SUPPLEMENTARY INFORMATION

Ageing and caloric restriction in a marine planktonic copepod

Enric Saiz[a,*], Albert Calbet[a], Kaiene Griffell[a], José Guilherme F. Bersano[a,b], Stamatina Isari[a,c], Montserrat Solé[a], Janna Peters[d], Miquel Alcaraz[a]

[a]Institut de Ciències del Mar – CSIC, Pg. Marítim de la Barceloneta 37-49, 08003 Barcelona, Catalonia, Spain

[b]Centro de Estudos do Mar, Universidade Federal do Paraná, Pontal do Paraná, PR, Brasil

[c]Institute of Oceanography, Hellenic Centre for Marine Research, P.O. Box 712, 19013 Anavyssos, Athens, Greece

[d]Institute for Hydrobiology and Fisheries Science, Hamburg University, Grosse Elbstrasse 133, 22767 Hamburg, Germany

[*]corresponding author: enric@icm.csic.es; Phone: 34+932309521; Fax: 34+932309555

SUPPLEMENTARY FIGURES

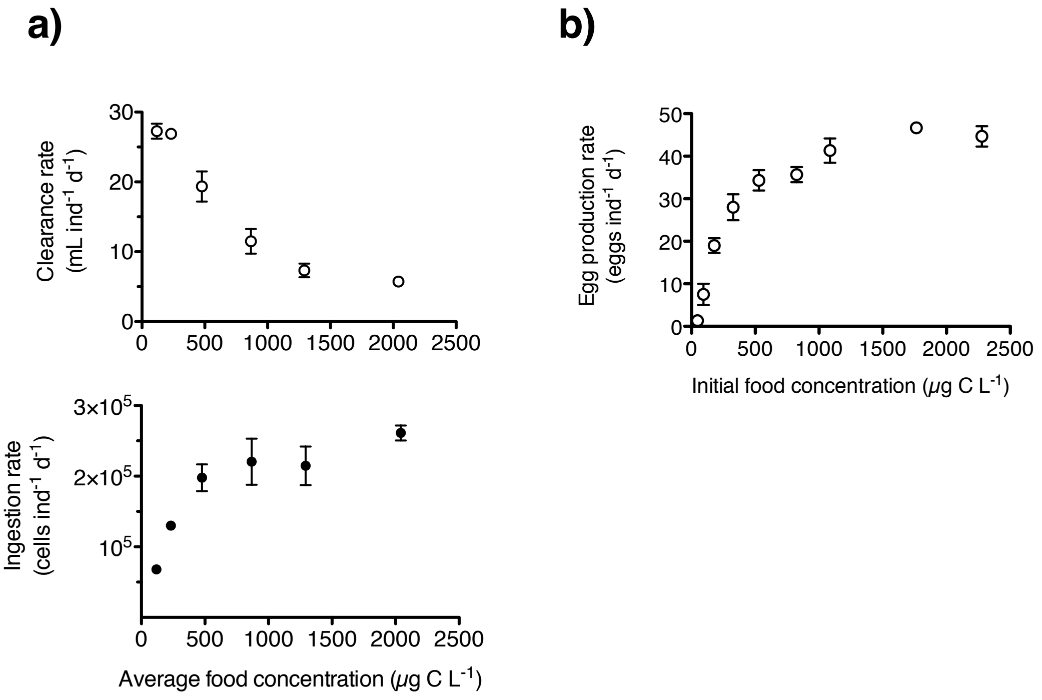

Supplementary Figure S1. Feeding (**a**) and egg production (**b**) functional responses of the copepod *Paracartia grani* as a function of the concentration of the alga *Rhodomonas salina*. Data in (**a**) correspond to unpublished feeding experiments conducted at 20ºC, following the methods in Isari and Saiz[1]. Data in (**b**) originates fom Calbet and Alcaraz[2], expressed in the original measurement unit of eggs ind^{-1} d^{-1}; those experiments were conducted at 18ºC.

REFERENCES

1 Isari, S. & Saiz, E. Feeding performance of the copepod *Clausocalanus lividus* (Frost and Fleminger 1968). *J. Plankton Res.* **33**, 715-728 (2011).

2 Calbet, A. & Alcaraz, M. Growth and survival rates of early developmental stages of *Acartia grani* (Copepoda: Calanoida) in relation to food concentration and fluctuations in food supply. *Mar. Ecol.-Prog. Ser.* **147**, 181-186 (1997).
